# John Raven, FRS, FRSE: a truly great innovator in plant physiology, photosynthesis and much more

**DOI:** 10.1007/s11120-025-01139-4

**Published:** 2025-02-17

**Authors:** A. W. D. Larkum, P. G. Falkowski, Dianne Edwards, C. B. Osmond, H. Lambers, P. Sanchez-Baracaldo, R. J. Ritchie, J. W. Runcie, P. J. Ralph, M. Westoby, S. Maberly, H. Griffiths, F. A. Smith, J. Beardall

**Affiliations:** 1 Climate Change Cluster, University of Technology Sydney, Building 7, Thomas St, Broadway, Ultimo, NSW 2009 Australia; 2 Institute of Marine and Coastal Sciences, Rutgers University, New Brunswick, NJ 08901 USA; 3 Biology, Cardiff University, Cardiff, CF10 3AT Wales; 4 Research School of Biology, Australian National University, Canberra, ACT 0200 Australia; 5 School of Biological Sciences, University of Western Australia, Perth, WA 6009 Australia; 6 School of Geographical Sciences, University of Bristol, Bristol, BS8 1TH UK; 7 Techology and Environment, Prince of Songkla University Phuket, Phuket, 83120 Thailand; 8 Aquation Pty Ltd, PO Box 3146, Umina Beach, NSW 2257 Australia; 9 Climate Change Cluster, University of Technology Sydney, Building 7, Thomas St, Broadway, Ultimo, NSW 2009 Australia; 10 School of Natural Sciences, Macquarie University, Macquarie Park, NSW 2109 Australia; 11 Centre for Ecology & Hydrology, Lancaster University, Lancaster, LA1 4YW UK; 12 Department of Plant Sciences, University of Cambridge, Cambridge, CB2 1TN UK; 13 University of Adelaide, Adelaide, South Australia 5005 Australia; 14 School of Biological Sciences, Monash University, Clayton, VIC 3800 Australia

**Keywords:** John A Raven: ion transport, Photosynthesis, Shoot-root interactions, Early land plants, extraterrestrial life, Algae

## Abstract

This is a tribute to a truly inspirational plant biologist, Prof. John A. Raven, FRS, FRSE (25th June 1941– 23rd May 2024), who died at the age of 82. He was a leader in the field of evolution and physiology of algae and land plants. His research touched on many areas including photosynthesis, ion transport, carbon utilisation, mineral use, such as silicon, iron and molybdenum, the evolution of phytoplankton, the evolution of root systems, the impact of global change, especially on the acidification of the oceans, carbon gain and water use in early land plants, and ways of detecting extraterrestrial photosynthesis. Beginning his research career in the Botany School, University of Cambridge, John studied ion uptake in a giant algal cell. This was at the time of great strides brought about by Peter Mitchell (1920–1992) in elucidating the role of energy generation in mitochondria and chloroplasts and the coupling of ion transport systems to energy generation. With Enid MacRobbie and Andrew Smith, John pioneered early work on the involvement of ion transport in the growth and metabolism of plant cells.On leaving Cambridge John took up a lectureship at the University of Dundee in 1971, where he was still attached upon his death. His primary focus over the years, with one of us (Paul Falkowski), was on phytoplankton, the photosynthetic microalgae of the oceans. Still, his publication list of 5 books and over 600 scientific papers spans a very broad range. The many highly cited papers (see Table 1) attest to an outstanding innovator, who influenced a multitude of students and coworkers and a very wide readership worldwide. At the personal level, John Raven was a wonderful human being; he had an extraordinary memory, dredging up facts and little-known scientific papers, like a scientific magician, but at the same time making humorous jokes and involving his colleagues in fun and sympathetic appreciation.

**Table 1:** Ten best cited articles (from google scholar)

	Citations	Date
Aquatic Photosynthesis, 3rd Edition P.G. Falkowski & J.A. Raven Princeton University Press, 2013	3854	2013
The evolution of modern eukaryotic phytoplankton P.G. Falkowski, M.E. Katz, A.H. Knoll, A. Quigg, J.A. Raven, et al Science **305**, 354–360	1790	2004
CO_2_ concentrating mechanisms in algae: mechanisms, environmental modulation, and evolution M. Giordano, J. Beardall & J.A. Raven Annu. Rev. Plant Biol. **56** (1), 99–131	1648	2005
Algae as nutritional food sources: revisiting our understanding M.L. Wells, P. Potin, J.S. Craigie, J.A. Raven, S.S. Merchant, et al Journal of applied phycology **29**, 949–982	1527	2017
Plant Nutrient acquisition strategies change with soil age H. Lambers, J.A. Raven, G.R. Shaver & S.E. Smith Trends in ecology & evolution **23**, 95–103	1488	2008
Ocean acidification due to increasing atmospheric carbon dioxide J. Raven, K. Caldeira, H. Elderfield, O. Hoegh-Guldberg, P. Liss, et al The Royal Society, Policy Document, June 2005	1470	2005
Phytoplankton in a changing world: cell size and elemental stoichiometry Z.V. Finkel, J. Beardall, K.J. Flynn, A. Quigg, T.A.V. Rees & J.A. Raven Journal of plankton research **32**, 119–137	1198	2010
Opportunities for improving phosphorus efficiency in crop plants E.J. Veneklaas, H. Lambers, J. Bragg, P.M. Finnegan, C.E. Lovelock, et al New phytologist **195**, 306–320	951	2012
Adaptation of unicellular algae to irradiance: an analysis of strategies K. Richardson, J. Beardall & J.A. Raven New Phytologist **93**, 157–191	914	1983
Nitrogen assimilation and transport in vascular land plants in relation to Intracellular pH regulation J.A. Raven & F.A. Smith New Phytologist **76**, 415–431	893	1976
Temperature and algal growth J.A. Raven & R.J. Geider New phytologist **110**, 441–461	867	1988
The role of trace metals in photosynthetic electron transport in O_2_ -evolving organisms J.A. Raven, M.C.W. Evans & R.E. Korb Photosynthesis Research **60**, 111–150	840	1999

Ten best cited articles (from google scholar)

Aquatic Photosynthesis, 3rd Edition

P.G. Falkowski & J.A. Raven

Princeton University Press, 2013

The evolution of modern eukaryotic phytoplankton

P.G. Falkowski, M.E. Katz, A.H. Knoll, A. Quigg, J.A. Raven, et al

Science **305**, 354–360

CO_2_ concentrating mechanisms in algae:

mechanisms, environmental modulation, and evolution

M. Giordano, J. Beardall & J.A. Raven

Annu. Rev. Plant Biol. **56** (1), 99–131

Algae as nutritional food sources: revisiting our understanding

M.L. Wells, P. Potin, J.S. Craigie, J.A. Raven, S.S. Merchant, et al

Journal of applied phycology **29**, 949–982

Plant Nutrient acquisition strategies change with soil age

H. Lambers, J.A. Raven, G.R. Shaver & S.E. Smith

Trends in ecology & evolution **23**, 95–103

Ocean acidification due to increasing atmospheric carbon dioxide

J. Raven, K. Caldeira, H. Elderfield, O. Hoegh-Guldberg, P. Liss, et al

The Royal Society, Policy Document, June 2005

Phytoplankton in a changing world: cell size and elemental stoichiometry

Z.V. Finkel, J. Beardall, K.J. Flynn, A. Quigg, T.A.V. Rees & J.A. Raven

Journal of plankton research **32**, 119–137

Opportunities for improving phosphorus efficiency in crop plants

E.J. Veneklaas, H. Lambers, J. Bragg, P.M. Finnegan, C.E. Lovelock, et al

New phytologist **195**, 306–320

Adaptation of unicellular algae to irradiance: an analysis of strategies

K. Richardson, J. Beardall & J.A. Raven

New Phytologist **93**, 157–191

Nitrogen assimilation and transport in vascular land plants in relation to Intracellular pH regulation

J.A. Raven & F.A. Smith

New Phytologist **76**, 415–431

Temperature and algal growth

J.A. Raven & R.J. Geider

New phytologist **110**, 441–461

The role of trace metals in photosynthetic electron transport in O_2_ -evolving organisms

J.A. Raven, M.C.W. Evans & R.E. Korb

Photosynthesis Research **60**, 111–150

## Career summary

John Albert Raven came from a farming family in north Essex. Early in the twentieth century, his grandparents moved their farm stock and dairy herd from Whitehaven (then Cumberland) to Wimbish in Essex. His parents, John Harold Edward Raven and Evelyn Falls (of Saffron Waldren) married in December, 1937. His cousin, Laura Mynott, recalls John being called home from primary school to round up escaped turkeys, and his interest in nature was revealed in an early letter as an 8-year-old to BBC Children’s Hour, on the longevity of sparrows. John’s secondary school was at the Friends’ School in Saffron Walden, and here, his interest in the workings of both crops and natural vegetation was deepened by an inspiring and suitably named teacher, Ken Plant.

**Fig. 1:**
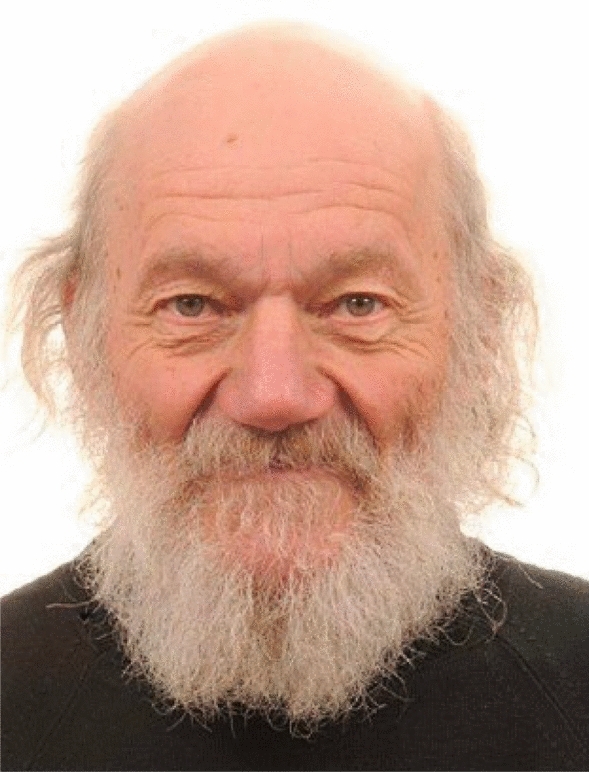
A portrait photo of John Raven taken in 2010

At the University of Cambridge (St John’s College), John specialised in plant physiology for the final year of his degree in Natural Sciences (Botany). After graduating in 1963, he undertook a PhD with Prof Enid MacRobbie (1931 – 2024), FRS. He was subsequently awarded a Junior Research Fellowship and a fixed-term lectureship. In 1971, he was awarded a lectureship in the Department of Biology, University of Dundee, where he was appointed to a Personal Chair in 1980, and was the John Boyd Baxter Professor of Biology from 1995 until his retirement in 2008. He continued working daily as part of the Life Sciences Division of the University at the James Hutton Institute, Invergowrie, and published over 600 scientific papers. At the same time, he held an Adjunct Professorship at the University of Western Australia, and a Visiting Professorship at the University of Technology Sydney. Throughout his academic career he was awarded many academic accolades, including Fellow of the Royal Society of Edinburgh (1981), Fellow of the Royal Society of London (1990), Doctor of Philosophy honoris causa by the University of Umeå in Sweden (1995), Highly Cited Scientist in Animal and Plant Biology by ISI (2002), Award of Excellence from the Phycological Society of America (2002), Honorary Life Membership of the British Phycological Society (2006), and Corresponding Member of the Botanical Society of America (2009). He is survived by his partner, and then wife, of 43 years, Linda Handley-Raven. A recent photograph of John is provided in Fig. 1.

John’s contributions to plant science are covered in separate sections below, and, personal reminiscences of his close colleagues, follow this.

### Cambridge, 1963–1971

**Fig. 2:**
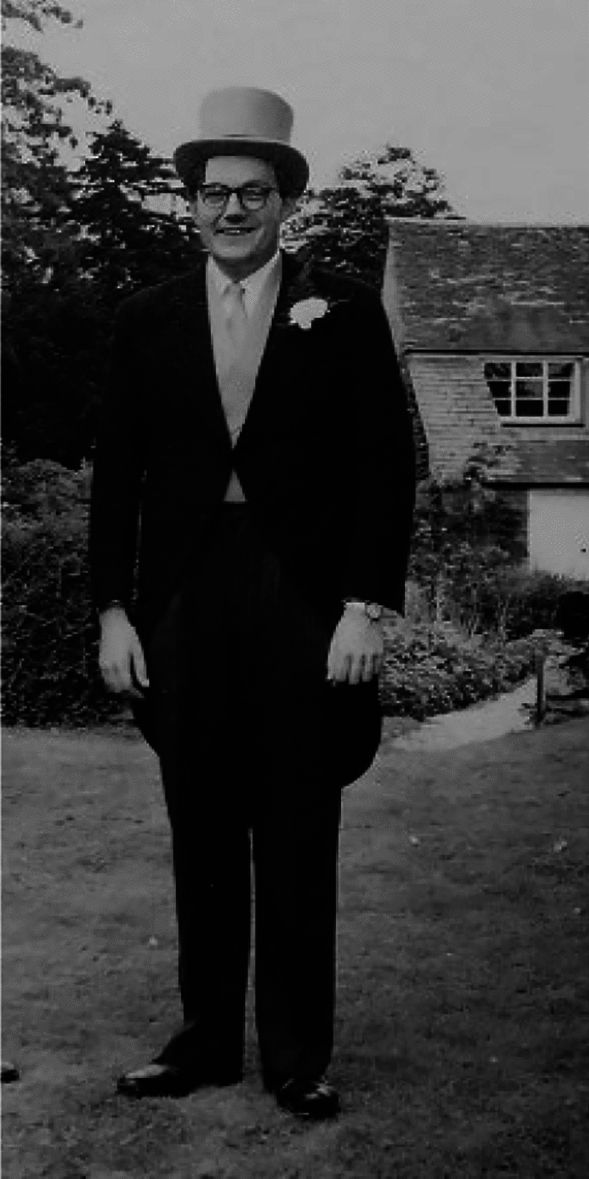
John Raven as best man at the wedding of Andrew Smith and Sally Harley in 1965

In 1958 Enid MacRobbie, who had finished a Ph. D.with Prof. Jack Dainty in Edinburgh, initiated a laboratory in the Botany School, Cambridge, to study ion transport in plant cells. This was at a time when plant physiology in the department was entering a low ebb, despite the energetic work of Tom ap Rees. Gone were the days of Bob Robertson and Michael Pitman, who had both returned to Australia, and G.E. Briggs was in retirement. Thus, the advent of Enid attracted many students and postdoctoral fellows; MacRobbie was an inspiring leader with wide-ranging interests; she was appointed to a Personal Professorship in 1987, the first woman scientist in Cambridge to be awarded a Personal Chair and was elected a Fellow of The Royal Society in 1991. The students in question were John (Albie) Raven, Andrew Smith, John Cram and Hugh Sadler, each of whom have left a solid legacy in the field of ion transport in plants. The postdocs were Roger Spanswick, Adrian (Sam) Hill, Ben Robinson, Barry Osmond (also with Tom ap Rees), Bruria Schachar-Hill, Mike Blatt, Dale Spender and one of the authors (Tony Larkum). Also in the lab was Fay Bendall, who formed a link to Derek Bendall and Robin Hill in the Biochemistry Department. On the periphery were Sally Harley, who married Andrew Smith (see photograph of John at Andrew’s and Sally’s wedding in 1965; Fig. 2), and carried out a PhD with Denis Garrett, on mycorrhizae (see below), and later made a strong connection to John Raven (see below) and Di Edwards, also to make a strong connection with John (see below). It was an exciting time!

John began his studies on the giant-celled alga, *Hydrodictyon africanum*. John measured ^14^C uptake over a range of solution pH as part of his PhD thesis, concluding that at high solution pH there must be HCO_3_^−^ uptake (Raven, PhD thesis, 1966). Andrew Smith working independently, as a postdoc, on four charophytes (*Nitella translucens*, *Chara australis* (aka *C. corallina), Nitellopsis obtusa* and *Tolypella intricata*) showed that there was light-driven HCO_3_^−^ uptake at high pH, all of which developed external calcification as a result of bicarbonate uptake (Smith 1967, 1968). John published a fuller account, including HCO_3_^−^ light-driven uptake in *Hydrodictyon* in 1968 (Raven 1968). It was the beginning of a collaboration with Andrew Smith, which continued on into the 1990s.

When Andrew Smith left for Australia in 1967, he formed a long-distance association (Adelaide – Sydney) with Alan Walker, who had previously been with Jack Dainty in the University of East Anglia, in Norwich. This forged a strong Australian connection, which continued until after the Covid pandemic in 2020, after which John was not allowed to fly. The earlier work showed that ion transport involved ATP-mediated active H^+^ efflux and passive OH^−^ influx coupled to, and a driver of, Cl^−^ influx (Smith 1970), and that osmotic control was a key factor. John recognised the importance of this work and collaborated in subsequent work (Raven and Smith 1973). These publications showed that intracellular (biochemical) processes alone cannot regulate cytoplasmic pH in plants, whether photosynthetic or otherwise, in the absence of plasma membrane H^+^- transport: hence the notion of a combined biochemical-biophysical pH–stat.

But we are getting ahead of the story. John moved to Dundee in 1971, to take up a lectureship in biological sciences—continued in the next section.

### Dundee, 1971–1980

After his move to Dundee in 1971, John Raven catalysed a cohort of postdocs and students to go on with the details of this work, employing a host of different algae, and later land plants. But a quick look at his publication list shows that John was at the same time publishing numerous single-authored papers on a multitude of topics.

During these early years at Dundee, John continued with studies on ion transport and its energetics, working mostly on the green alga *Hydrodictyon*. Importantly he further developed, in collaboration with Andrew Smith, ideas about pH regulation and H^+^ transport in algal and land plant cells. This culminated in a series of influential papers showing how cells control cytoplasmic pH to facilitate ion exchange across cell membranes (see e.g. Raven and Smith 1976a; Smith and Raven 1979). In the latter part of the 1970s John, with postdocs Sue Allen, and Maria De Michelis, further probed these ideas with studies on consequences of nitrogen source (nitrate vs ammonia) for internal pH regulation in the castor oil plant, *Ricinus* (Raven and Allen 1983) and in *Hydrodictyon* (Raven and De Michelis 1979, 1980) and this was a theme John would continue to explore in subsequent decades.

At this time John also worked on on transmembrane fluxes of the plant growth regulator, auxin, indole acetic acid (IAA) in *Hydrodictyon* (Raven 1975). In doing so, he recognised the chemiosmotic basis to polar auxin transport, insights which complemented the work of Rubery and Sheldrake (1974) and made a major contribution to our understanding of auxin movement and apical dominance in vascular plants. In the late 1970s also see John’s first forays into ideas on cell size and allometry, which would continue throughout his career (Raven and Smith 1977; Raven et al. 1979). Evolution was a thread ramifying through most of John’s interests and some of the earlier work on this appeared in the 1970s in relation to evolution of chemiosmosis (again with Andrew Smith) (Raven and Smith 1976b) and on evolution of structure and function in land plants (Raven 1977), the latter giving rise to a later collaboration with Dianne Edwards (see below).

A major component of Raven’s interests in the 1970s was carbon assimilation in algae. Following on work he had begun while at Cambridge, John contributed, with postdoc Sheila Glidewell, seminal work (again in *Hydrodictyon*) on photorespiration (Glidewell & Raven 1975, 1976) as well as reporting that green algae showed physiological characteristics of C_4_ photosynthesis (low CO_2_ compensation points, high affinity for CO_2_ and low inhibition by O_2_) similar to those of terrestrial C_4_ plants, despite having Rubisco as the primary enzyme of carbon assimilation (Raven & Glidewell 1978). These characteristics were explained by the work of Aaron Kaplan and Murray Badger in Joe Berry’s lab in the Carnegie Institute in Washington, USA, showing directly that *Chlamydomonas reinhardtii* and *Anabaena variabilis* accumulated CO_2_ intracellularly in a process known as a CO_2_ concentrating mechanism (CCM), which became a major research focus of John, his students and postdocs.

Subsequent work in Raven’s laboratory suggested that CCMs were likely driven by active transport of bicarbonate, probably at the chloroplast envelope and that this led to lower (i.e. more positive) carbon isotope discrimination values. From this time, Raven pioneered, with Graham Farquhar, in Canberra, Australia, and later with his wife, Linda Handley-Raven, the use of carbon isotope discrimination as a tool for investigating the distribution and activity of CCMs and bicarbonate use versus diffusive CO_2_ entry in algal photosynthesis (Raven and Farquhar 1990).

The 1970s was a decade that saw the genesis of many of John Raven’s ideas (and there were many!) which later saw the light of day in an astonishing number of publications.

### The 1980s

John Beardall had joined John Raven’s lab as a postdoc in 1979. He had previously held a postdoc with G.E. (Tony) Fogg at University College of North Wales in Menai Bridge doing field work on phytoplankton ecology in Liverpool Bay. Working with John offered exciting opportunities to follow up some of his PhD work on inorganic carbon assimilation,

With this background and the work on CCMs in Joe Berry’s group (at the Carnegie Institute of Washington at Stanford University), John Beardall, joined John’s group, to look at aspects of CO_2_ concentrating mechanisms and their modulation by environmental conditions. Working on *Chlorella*, they showed that, when CCMs were operative, changes in membrane potential were consistent with active primary transport of bicarbonate, most likely at the chloroplast envelope (Raven and Beardall 1981, Beardall & Raven 1981). John Beardall and Howard Griffiths (working on a PhD in the lab of Janet Sprent, next door,) showed that CCMs resulted in lower (i.e. more positive) carbon isotope fractionation values and that nitrogen limitation induced CCM activity, even at high CO_2_ (Beardall et al. 1982). They also worked on C acquisition in freshwater macrophytes and demonstrated, amongst other things, that the capacity to use exogenous bicarbonate rather than CO_2_ also altered isotope fractionation values (Raven et al 1982a, b).

The use of carbon isotope fractionation values as a tool for investigating the distribution and activity of CCMs and bicarbonate use versus diffusive CO_2_ entry in algal photosynthesis was to be a persistent theme in John’s later work. Work on inorganic carbon acquisition continued into the 1980s (and indeed was a strong theme throughout John’s career) with work in freshwater macrophytes (Raven & Beardall 1981; Raven et al 1982a, b) and marine macroalgae. The late 1970s saw John’s first forays into supervising PhD students: Andrew M. Johnston (working on inorganic carbon acquisition in macroalgae; PhD awarded 1982); Mitchell Andrews (working on ecology and physiology of *Chara hispida;* PhD awarded 1984); Misni bin Surif and Howard Griffiths (supervised by Janet Sprent, but also overseen by John)) were John’s first.

This wonderful collaboration with Beardall continued until John’s sudden death in May 2024 and covered topics from the role of primary active transport in algal energetics (Raven and Beardall 2020), via evolution and modulation of CCMs (e.g. Giordano et al. 2005) to the influence of global climate change on plankton (Raven and Beardall 2021). Indeed, it was only a few weeks before his passing that John had finished helping check page proofs of a book edited and contributed to by himself, Mario Giordano, Stephen Maberly and John Beardall (Giordano et al. Giordano Beardall Maberly Raven 2024). John was always extraordinarily generous in involving colleagues in collaborative work (the list of his co-authors is enormous!) and his influence on generations of up-and-coming biologists was immense.

It was in the 1980s that John really got into his stride and published on an incredibly diverse range of subjects. During this decade he published over 50 papers; the topics being: light and temperature effects on algal growth; ion transport in algae; pH regulation in algae and vascular plants; the role of silicon, iron, molybdenum and boric acid in algae; the role of vacuoles in algae; the primary productivity of a variety of brown algae; the role of unstirred layers and relationships to Rubisco activity; photoinhibition; the uptake of CO_2_ especially in the roots of Isoetids; the energy costs of carbon acquisition; the evolution of early land plants; the evolution of nitrogen-fixing symbioses; photosynthesis in diatoms and dinoflagellates; biomineralization in characeans; and the movement of viruses in terrestrial plants.

In the above-noted work John was aided by many PhD students, postdocs and visitors: these included, in addition to those mentioned above, Richard Geider, Jeffrey Macfarlane, Andrew Miller, Bruce Osborne, Barruy Osmond and Göran Samuelsson. At this time, too, John shared a lab with another lecturer, Janet Sprent; and they published multiple papers together.

**Fig. 3:**
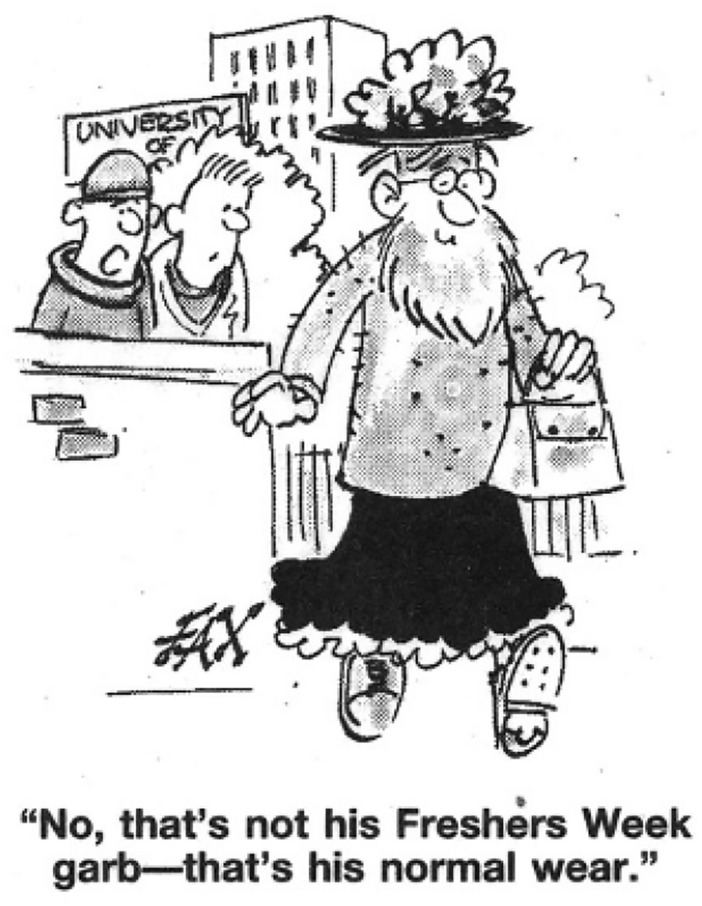
A cartoon that appeared in the Dundee Courier, 1999; courtesy Ali Rob

John also initiated his world-wide travels at this time, especially to Australia; to the laboratories of Barry Osmond, John Beardall, Alan Walker and Andrew Smith. Bruce Osborne has published separately, in an obituary for John Raven, on the original proposal of Jon Keeley in the 80 s that in *Isoetes* plants there is a CAM fixation pathway in the roots. This attracted John’s attention, even though it was not fashionable. With Barry Osmond, John collected plants in the Andes (see Fig. 3) and back home in Scotland obtained convincing evidence that Isoetids and close relatives did indeed have a CAM fixation mechanism in their roots: Keely et al. (1984).

Two of his ten best cited papers (Table 1) come out of this period: “*Adaptation of unicellular algae to irradiance: an analysis of strategie*s” by Richardson, Beardall & Raven (Raven and Allen 1983); and “*Temperature and algal growth*”, by Geider and Raven (1988). Other papers are still high on anybody’s list. such as “The role of vacuoles” (Raven 1987). The latter was a magisterial work of 65 pages. Like many of John’s papers it must have been a long time in the making. It covered a great deal of new ground. For instance, it covered the topic of increased light harvesting in multicellular plants with vacuolated cells: in, for example, many seaweeds, especially the green algae and their successors the land plants. But John went on from there to formulate the cost–benefit of having vacuolated cells; a subject that was to recur many times subsequently (e.g. Raven 1997). And then he turned this on its head and asked the question, what benefit it was to have no vacuole, as in the picoplankton. And this was another theme that was to recur in many subsequent papers.

In addition, John published a well-cited book “Energetics and transport in aquatic plants,” A.R. Liss, NY, 1984.

A photograph of John’s office is shown in in 1991, in Fig. 3. This was before the advent of computers and data bases, of which the latter caused John some tribulation (see Reminiscence of Tony Larkum).

### The 1990s

John published over 70 papers in the 1990s, and just to read through the range of subjects is bewildering. He started off the decade with papers on “*Mn and Fe use efficiency in phototrophs*” (Raven 1990a, b), then “*The influence of N metabolism and organic acids on the natural abundance of carbon isotopes*” (Raven and Farquhar 1990), then “*Sensing pH*” (Raven 1990a, b), and “*Carbon metabolism*” (Raven & MacFarlane, 1990), and “The growth of *Fucus in high CO*_*2*_ “ (Johnson and Raven 1990), just for starters.

During the ensuing years there was a steady stream of work on algae in the marine waters of Scotland – red algae with Janet Kubler, Johnston and Stephen Maberly, *Enteromorpha* with L.J. Poole, and the usual list of green algae. This work also included members of the plankton, such as diatoms and the picoplankton. He kept up a keen interest on CO_2_ uptake mechanisms in algae, together with John Beardall, but began to consider land plants as well; this led on to several papers on the interpretation of physiological constraints imposed on gas exchange and water use efficiency as determined by xylem, air spaces and the evolution of stomatal structures, as elegantly visualised with the exquisite fossil images of Prof. Di Edwards: e.g. *The influence of N metabolism and organic acid synthesis on the natural abundance of isotopes of carbon in plants* (Raven and Farquhar 1990). *Discrimination between C*_*12*_* and C*_*13*_* by marine plants* (Maberly et al. 1992); *Into the voids: the distribution, function, development and maintenance of gas spaces in plants* (Raven 1996); *The *^*15*^*N natural abundance (δ *^*15*^*N) of ecosystem samples reflects measures of water availability* (Handley et al. 1999); *Roots: evolutionary origins and biochemical significance.* (Raven and Edwards 2001).

The long series of papers with Linda Handley-Raven, stretching right up to the time of his death, began at this time, including those on topics, such as “*Transport processes and water relations*” (Raven and Handley 1987); “*Ammonia and ammonium fluxes between photolithotrophs and the environment in relation to the global nitrogen cycle*” (Raven 1996, 1992); “*A comparison of NH*_*4*_* and NO*_*3*_* as a source for photolithotrophs*” (Raven 1996); “*The use of natural abundance of nitrogen isotopes in plant physiology and ecology*” (Handley et al. 1997, 1992); and “*Chromosome 4 controls potential water use efficiency (*δ ^*13*^*C) in barley*” (Handley, Nevo and Raven, Handley et al. 1994).

One of John Raven’s greatest interests was in evolution, and this led to research into how different mechanisms of ion uptake had been applied at various stages of the evolution of cyanobacteria into the plastids of early algae, and then on to the ancestors of land plants, leading on to the conquest of the land and the evolution of land plants up to flowering plants. John was interested in the most difficult of questions, such as how land plants would maintain nutritional status between roots in the ground and aerial stems and leaves above-ground. John first tackled the question of the root-shoot balance for acid–base relations in a paper in 1988 (Raven, 1988). And later in association with Dianne Edwards, he worked out how the acid and ion balance would have been maintained to give optimal nutrition (Raven & Edwards 2001, Edwards and Raven, 2004, Raven and Edwards 2004, Edwards, Raven and Edwards, 2013, Cherne and Raven 2011, 2015).

The very popular book “Aquatic Photosynthesis”, authored by Paul Falkowski and John Raven, first came out in 1996. This was the result of several years of collaboration; and the background as to how this book came about is described in a reminiscence by Faul Falkowski (see below). It went on to three editions. In addition, it was accompanied by many highly cited papers between the John and Paul: on the evolutionary strategies of the phytoplankton of the oceans (see the Twenty-First Century, below)).

Of special interest at this time was the evolution of the world’s phytoplankton, of which the diatoms, the dinoflagellates and the green algae, were the central, but by no means the only, players. The initial idea on the evolution of phytoplankton in the World’s Oceans (light utilization, allometry and elemental stoichiometry) seems to have come about by the interaction of Zoe Finkel and Antonietta Quigg. Zoe Finkel published a seminal paper in 2001 (Finkel 2001) from the Department of Biology at Dalhousie University, Canada, on “*Light absorption and size scaling of light-limited metabolism in marine diatoms*”. This, of course, was a subject close to John’s heart and led to an early paper (Raven & Kübler 2002). Antonietta Quigg had joined John Beardall’s laboratory to carry out a PhD. in the late 1990s, and then joined Paul Falkowski as a postdoc in early 2000s leading to a several seminal papers: “*The evolution of modern eukaryotic phytoplankton*” (Falkowski et al. 2004); “*Irradiance and the elemental stoichiometry of marine phytoplankton*” (Finkel, Quigg, Raven et al., 2006); followed by important papers by Raven, Falkowski and others, especially in “*Evolution of Primary Producers in the Sea*”, edited by Paul Falkowski and Andrew Knoll in 2007 (published by Academic Press). The idea of scaling in large and small (pico) plankton was a subject that John Raven continued to explore in the 2000s (see below).

**Fig. 4:**
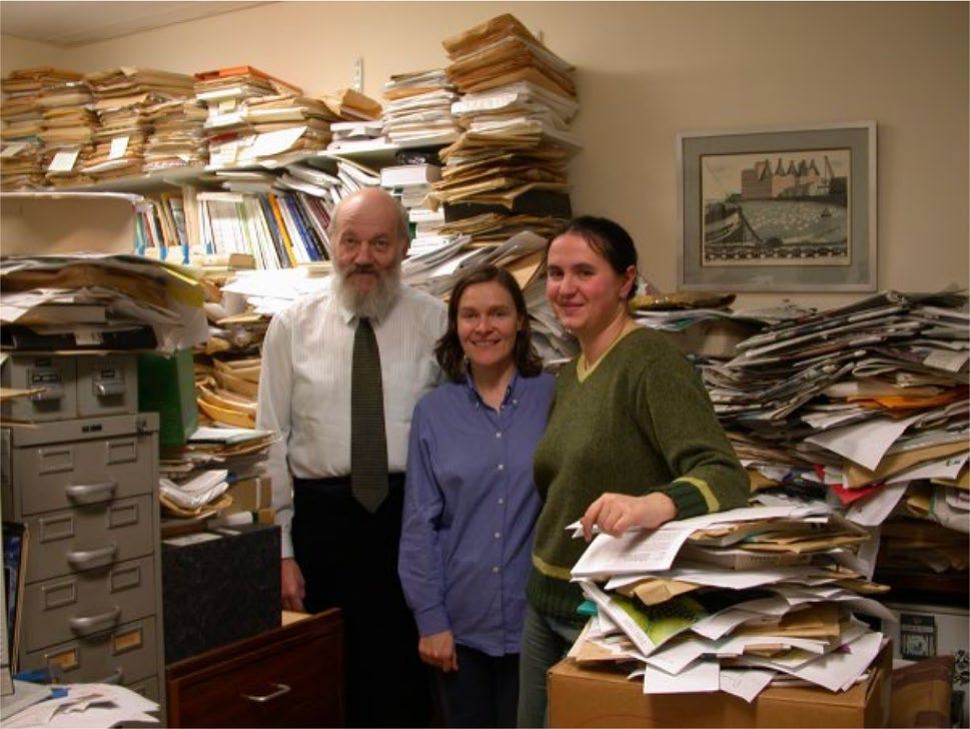
John Raven in his office at Dundee University in 1991 with students Alessandro Norici and Shona McInroy. Courtesy of Alessandro Norici. Eventually John employed a secretary to transfer much of his reference collection into digital form. Despite the formidable look of his office, John was able to access any reference within a few minutes, even including the grey literature

It was in the 1990s that John took to wearing a kilt and this was recognised by a local Dundee newspaper – see Fig. 4.

### The twenty-first century

**Fig. 5:**
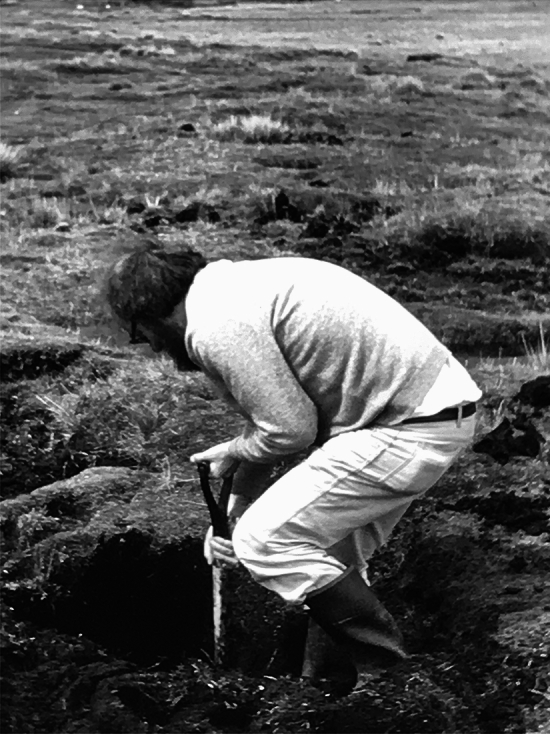
In the photo of John Raven is shown exhuming the root system of *Stylites* from peat at high elevation (> 4000 m) in the foothills of the Peruvian Andes in 1982–3. It was digitised from a colour slide I took during the field trip organised by Jon Keeley that resulted in the *Nature* paper. Cornelia Büchen-Osmond scanned and sharpened the image. Keeley, J.E., Osmond, C.B. and Raven, J.A. (1984) *Stylites*, a vascular land plant without stomata absorbs CO_2_ via its roots. Nature 310, 694–695

While there was no abatement of this singular stream of original contributions in the twenty-first century, the general themes remained the same, with the addition of a few new themes such as extra-terrestrial life and photosynthesis. John travelled often to Australia forming associations with Hans Lambers in Western Australia, and Peter Ralph, Mark Westoby and Tony Larkum in Sydney Fig. 5.

While John continued with his regularly list of subjects – CO_2_ supply, nutrient relations (Raven et al 2002a, b), nitrogen fixation (Raven et al. 2004); evolution of photosynthesis (Beardall and Raven 2004; Giordano et al. 2005; Raven et al. 2008)), small is beautiful and picoplankton (Raven et al. 2005a, b, c, 2013).

– he branched out into new directions:

1. Ocean acidification (Raven et al 2005a, b, c),
2. The cost of photoinhibition (Raven 2011),
3. Cell size and stoichiometry of elemental uptake (Finkel et al. 2010),
4. Opportunities for improving productivity in crop plants (Veneklaas et al. 2012),
5. Phosphate limitation of nitrogen-fixation in the planktonic cyanobacterium *Trichodesmium* (Saluda et al, 2001),
6. Pluses and minuses of ammonium and nitrate uptake and assimilation by phytoplankton, & its implications for productivity (Glibert et al. 2016),
7. Plant mineral nutrition in ancient landscapes & high plant species diversity (Lambers et al. 2011),
8. Root evolutionary origins and biogeochemical significance for the primary productivity of Planet Earth (Raven and Edwards 2001: Geider et al., 2001),
9. Potential effects of global climate change on algal photosynthesis (Beardall & Raven 2004).
10. Algae as a food and biofuel source: “Enhanced biofuel production” (Raven and Ralph 2015; “*Algae as nutritional and functional food sources: revisiting our understanding*” (Wells et al. 2017;).
11. Blue carbon science (Larkum et al. 2017; Raven 2018; Macreadie et al. 2019).
12. Extraterrestrial life and photosynthesis and their detection (Wolstencroft and Raven 2002; Cockell and Raven 2004; Cockell et al. 2009).

**Fig. 6:**
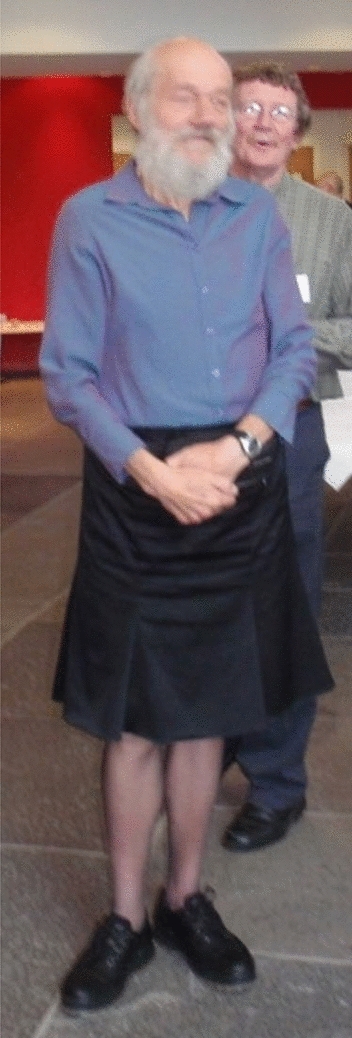
John Raven at the Symposium on Algae, Photosynthesis and Global Change held on 12th December 2008 at the University of Dundee to mark his official retirement. John is wearing his habitual clothes, a skirt for a kilt and a shirt-top, which often morphed into a blouse

These examples necessarily fail to acknowledge all the wonderful contributions and ideas that John put into his over 100 publications in the twenty-first century. On 12th December 2008 a Symposium on Algae, Photosynthesis and Global Change was held at the University of Dundee to mark Johns’s official retirement. Figure 6 shows John at this Symposium wearing what was by this time traditional garb.

Fittingly a posthumous publication of “Evolutionary Physiology of Algae and Aquatic Plants” (Giordano et al., Giordano Beardall Maberly Raven 2024) contains many contributions from John Raven. We must feel grateful for this surviving legacy, while decrying the loss of such a productive scientist.

### Reminiscences by some of John’s Friends

#### Dianne edwards, FRS, FRSE

My dominating aim as a plant-based palaeontologist has been to reconstruct ancient fossils as living and reproducing plants with a concentration on those that first colonised the land and, in their activities, changed the face of planet Earth. This has been achieved to a great extent by collaboration and consultation with John Raven, which began in postgraduate days in Cambridge, 60 years ago, and persisted through our disparate careers in Dundee and Cardiff. Our joint papers emphasise his amazing capacity to collate, assess and quantify earlier and current researches, including his own, to answer major evolutionary events. This was based on his encyclopaedic knowledge of organisms, ranging from cyanobacteria to embryophytes, embracing the morphology of thallophytes, bryophytes and tracheophytes, while at the same time explaining the nature of the functional and biochemical adaptions that were central to the colonisation of a hostile, water-stressed terrestrial environment. His interests had begun in benchmark papers first in 1984, ‘*Physiological correlates of early vascular plants*’ then superbly summarised in 1997, in ‘*The evolution of vascular plants in supracellular transport processes*’. These seminnal papers were followed by a series of papers in the 1990s in which the nature of homoeohydry and poikilohydry were explored in depth, the former in the evolution of conducting systems and control of water-loss via tracheids and stomata, with the often overlooked air space systems (See review in Raven and Edwards 2004).

Such a focus led to a consideration of the costs of photosynthesis and water-use efficiency in the context of the colonisation of the land, both at the level of the individual and up to global scales (see review in Raven and Edwards 2014) and encompassing future increases in greenhouse gases. These accumulated empirical data on extant analyses again covered a wide range of taxa relevant to early land plants, including variation in photosynthetic capacities relating to morphology and process that are invaluable to those interested in effects of early vegetation, sensu lato, on atmosphere and substrates.

Roots, as absorbing and anchoring structures, were not present in the earliest tracheophytes, their functions replaced, somewhat inefficiently, by rhizoids on horizontal and partially embedded aerial stems/axes. Limited evidence suggests that early lycophytes possessed adventitious branching root like structures, but lacked root hairs, root caps and endoderms. John had become interested in models of global atmospheric CO_2_ concentrations, for example Robert Berner’s GEOCARB II models, and implications for the role of roots in chemical weathering of rocks in the early drawdown of CO_2_ and global climate change. Roots would have fractured rocks, and in addition the microbial interactions would have been profound Drawdown of CO_2_ could well have decreased atmospheric temperature leading even to global glaciations, with a change from global greenhouse to icehouse Earth in the mid-Palaeozoic. Such hypotheses remain very much in vogue today.

In ‘*Could land-based early photosynthesising ecosystems bioengineer the planet in mid-Palaeozoic times?*’ (Edwards et al. 2015), evidence was documented for the palaeobotanical evidence of plant types, and concentrated on pre-vascular plant rooting communities that today are called cryptogamic covers (CCs) and include Cyanobacteria, Archaea and bacterial mats, fungi, bryophytes and lichens. This was complemented by John’s contributions: he systematically considered interactions of the cryptogamic covers with abiotic factors relevant to their growth, survival and their environmental impacts, including effects *on* and *of* CCs. He covered photosynthetically active and UV radiation, temperature, surface roughness, albedo, nutrients and ‘nutrient’ chemicals that regulate the physical environment including water, oxygen and carbon dioxide, as well as nitrogen; phosphorus and iron. In his estimates of net primary productivity biomass and biological nitrogen fixation as a basis for modelling, he warned of over concentration on extant tracheophyte-dominated systems, As usual, John advised on future research directions, and as with so many of John’s thought processes, these will challenge us, but broaden our horizons.

#### Paul Falkowski

I met John in the 1980s at various conferences, but in the early 1990s, we attended one on photosynthesis at the University of Hawaii in Manoa. I taught there each summer, and John visited frequently because his future wife, Linda, lived in Honolulu. Regardless, one of the speakers gave a talk about photosynthesis-irradiance curves. I had written several articles about the biophysics of the response of photosynthesis to irradiance, and the speaker was clearly confused and confusing. I poked John, who was sitting next to me, and whispered something to the effect, “We should write a textbook on photosynthesis”. He nodded and kept writing something or other on his pad. He was a compulsive writer.

A few months later, I outlined a book, sent the outline to John, and he and I began to write. John’s writing style and mine were completely different. By way of analogy, John wrote more like James Joyce–long sentences. I wrote more like Hemmingway–short sentences. Regardless, after about two years, our book, **Aquatic Photosynthesis**, came together. I wrote several chapters on the light reactions, with (what I think) are minimal equations. John wrote a chapter on the dark reactions and together we wrote chapters on evolution, and global photosynthesis. I rewrote most of John’s chapters/contributions, to keep the entire book in one voice. Originally published by Blackwell, in the UK, the book sold very well, for such a dense text, and we both were happy at its reception.

During the following decade, John and I worked on several papers together and then we revised the book. The second edition was published by Princeton University Press, and also had a good reception. By then, it had become a “classic” in the field.

John and I spent many hours together, discussing many things. His lab/office in Dundee was a fire hazard with all his papers piled up across lab benches. But he knew where every paper was and could find it in a few moments. His knowledge of the literature was exceptional. Indeed, he remembered papers from the 1930s in detail. It was an incredible pleasure to work with him – and to have a glass of wine at the end of the day, while we shared stories.

Beyond any doubt, John was an extraordinary person. I stayed at his home in Invergowrie, near Dundee, with Linda several times and he stayed at our place in Stony Brook, New York. He had a great sense of humour and would get up early to run before breakfast. In the end, he was an outstanding scholar and colleague.

#### Hans Lambers FAA

What impressed me most about John was his encyclopaedic knowledge. A good encyclopedia does not simply contain a lot of information, but has that information organised in specific sections with numerous cross references to other relevant sections. That is also how John’s brain functioned, in my experience. When I asked John to read one of my papers, he would often add a new dimension that I had not considered, and I would then offer him a co-authorship. The best example is a review published in 2010 (Lambers et al. 2011), on nutrional problems for plants in several Western Australian ecosystems; John pointed out the quantitatively important contribution of ribosomal RNA to leaf phosphorus concentrations. That laid the foundation for a very fruitful collaboration with Mark Stitt’s group (Lambers et al. 2012; Sulpice et al. 2014). Very fruitful interactions arose from workshops, one at the University of Western Australia, Perth (Veneklaas et al. 2012), and another hosted by Mark Westoby at Macquarie University in Sydney, which led to two influential papers (Lambers et al. 2008; Raven et al. 2018).

During the last few months of John’s life, we were collaborating on a manuscript on the growth rate hypothesis (Elser et al. 2003, 2010). Plant ecologists are very keen on using this hypothesis to explain scaling relations in terrestrial plants, involving leaf nitrogen and phosphorus concentrations (Kerkhoff et al. 2006). In John’s and my view, this hypothesis makes no sense when it comes to mature leaves of plants, because these leaves stopped growing. Together with Daniil Scheifes we were working towards an alternative explanation to explain the findings on scaling of nitrogen and phosphorus. If we can retrieve the text John wrote from his laptop, he will end up as a co-author on our opinion paper. If that turns out impossible, we will dedicate our paper to him, because we have been discussing this topic over a glass of wine on numerous occasions over the years, when John stayed at our place in Australia.

#### Stephen Maberly

I started my PhD at St Andrews, Scotland in 1978 with David Spence on the interaction between phytoplankton and macrophytes, particularly in connection with competition for inorganic carbon. Of course John was nearby at Dundee and I had read his papers on carbon uptake by *Hydrodictyon* (e.g. Raven 1968) and his influential review (Raven 1970) that has been cited over 300 times and is still being cited. I first met John at some stage during my PhD and had the opportunity to do a postdoc with him but opted instead to take on a more ecological topic with Jack Talling FRS at Windermere. Nevertheless, John and I corresponded but it was not until the late 1980s/early 1990s that we worked together. We studied the carbon isotopic composition of marine macroalgae with Andy Johnston, and showed that species restricted to carbon dioxide as a carbon source can be distinguished from those that access bicarbonate by their more depleted ^13^C content (Maberly et al. 1992). We co-supervised a Ph.D. student, Lucy Ball, who studied as many freshwater chrysophytes as she could obtain: all lacked the ability to use bicarbonate (Maberly et al. 2009). John went on to write a paper, one of his favourites apparently, on these and other species that lack a CCM (Raven et al. 2005a, b, c). Subsequently, John and I worked on various book chapters and journal reviews, largely motivated and led by John (e.g. Raven et al. 2012). Most recently, along with John Beardall and initially the late Mario Giordano, we completed editing a book for Cambridge University Press just before John passed (Giordano Beardall Maberly Raven 2024).

Everyone recognises that John was an amazing person in many ways. It might not be a description that John would welcome, but he was the only person I have met who could be called a genius. His knowledge was extremely broad, but not just scientifically. One evening at my house Lucy Ball and John discussed the wingspans of different passenger aircraft, a strange example of John’s encyclopaedic knowledge! His knowledge was also incredibly deep: when we discussed topics I thought I knew a lot about, I realised that John knew more about them than I did. John made a major contribution to a very wide range of different scientific topics including astronomy, oceanography, aquatic biology and ecology, plant sciences, evolution and biochemistry. At the same time he was a very modest and kind person: I rarely saw him criticise people at meetings (even though sometimes criticism would have been warranted). He was also loyal and supportive and helped me and many others in their careers. It was an honour to have known and worked with John and a pleasure to have received so many postcards from around the world. It was a fortunate chance that, because I was in the area, I met John and his supportive wife Linda at their house in Invergowrie, Dundee in early May 2024.

#### Barry Osmond, FAA, FRS: recollections aided by Cornelia Büchen-Osmond

For this stumbling antipodean in the Botany School Cambridge UK 1966–67 it was a treat to take a glass of cider with Tom ap Rees’ court in the pub at day’s end and there to meet John Raven, then one of Enid MacRobbie’s team of astonishingly sharp aspirants in plant biology.

John and I met often during a later sabbatical in the UK in 1980 when, memorably, he explained that if one hoped to understand boundary-layer effects on δ^13^CO_2_ fractionation in submerged aquatic plants, more accurate flow rates of water in streams would to be obtained with an orange than with a pooh stick!

John was already a frequent visitor to Australia when we sat together at the International Botanical Congress in Sydney (1981) and remarked on the absence of stomata on cuticles of fossilised early land plants during an authoritative plenary.

John Keeley overheard our discussion and immediately volunteered a field excursion to the high Andes of Peru that was worth every bit of the headaches from shovel work in peat at 4500 m elevation, and the cover photo for.

Keeley et al., (1983). (John Keeley’s wife Stirling had the last word: “How does *Stylites* CAM-peat”?

Some 20 years later Cornelia and I were delighted that John and Linda might become visiting faculty at the embryonic Biosphere 2 campus of Columbia University in Oracle Arizona. Students had been delighted to have a tartan and sporran sported on campus but Columbia wanted to vet John’s CV. So I will wrap up our homage with some of the excerpts from this Columbia 63 page document:

**“John Raven has hundreds of peer reviewed publications, with 29 published or in press 2001-2, including………”**

His CV then listed publications in 79 different journals (including *Biofouling!*).

The Columbia document noted his examination and assessment of undergraduate and postgraduate courses and theses from 36 universities and the reviewing of research proposals for 20 different granting agencies around the world.

The summary was simply:

**“John Raven is one of the most widely read researchers in plant sciences. His familiarity with, and insightful contributions to, plant sciences in the broadest sense are reflected in the courses he has taught and in the lectures he is invited to give throughout the world. Not much in plant biology, evolution and environment escapes an authoritative opinion from John Raven.”**

Of all the “might have been” that was trashed in Columbia’s premature closure of the facility in 2003, John and Linda’s potential engagement had become a top priority.

**John Raven was the preeminent Polymath of our discipline; the**

**plant biology world wide is the poorer today for his passing**

**“Dear Linda: our sincere, deepest sympathy; our thoughts are with you.**

**We shall miss John’s postcards from all corners of the planet…….”**

**John Beardall**

I joined John Raven’s lab as a postdoc in 1979. I had previously held a postdoc with G.E. (Tony) Fogg at University College of North Wales in Menai Bridge doing field work on phytoplankton ecology in Liverpool Bay, which was a challenge given my susceptibility to seasickness. Working with John offered exciting opportunities to follow up some of my PhD work on inorganic carbon assimilation, all without the need to be at sea for one week every month!!

The 1970’s were exciting years to be working on carbon acquisition in algae. John had previously investigated bicarbonate vs CO_2_ as sources of inorganic carbon for photosynthesis in *Hydrodictyon africanum* (Raven 1968) and in a subsequent review (Raven 1970) he first raised the problem that the apparent K_m_ for CO_2_ of Rubisco is much higher than the exogenous [CO_2_], despite the very early reports (e.g. Steeman-Nielsen & Jensen 1958) that green algae such as *Chlorella* saturate photosynthesis at very low CO_2_ levels. In the mid-1970s he contributed, with postdoc Shelia Glidewell, seminal work (again in *Hydrodictyon*) on photorespiration (Glidewell & Raven 1975, 1976) as well as reporting that green algae showed physiological characteristics of photosynthesis (low CO_2_ compensation points, high affinity for CO_2_ and low inhibition by O_2_) similar to those of terrestrial C_4_ plants, despite having Rubisco as the primary enzyme of carbon assimilation (Raven & Glidewell 1978). Around this time, Aaron Kaplan and Murray Badger were working as postdocs in Joe Berry’s lab at the Carnegie Institute in Washington and showed directly that *Chlamydomonas reinhardtii* and *Anabaena variabilis* accumulated CO_2_ intracellularly (Kaplan et al. 1980, Badger et al. 1980). It was against this background that I joined John’s group to look at aspects of CO_2_-concentrating mechanisms (CCMs) and their modulation by environmental conditions. Working on *Chlorella*, we showed that changes in membrane potential when CCMs were operative were consistent with active primary transport of bicarbonate, most likely at the chloroplast envelope (Beardall et al. 1982, Beardall & Raven 1981). With Howard Griffiths, we showed that CCMs resulted in lower (i.e. more positive) carbon isotope fractionation values and that nitrogen limitation induced CCM activity, even at high CO_2_ (Beardall et al. 1982). We also worked on C acquisition in freshwater macrophytes and demonstrated, amongst other things, that the capacity to use exogenous bicarbonate rather than CO_2_ also altered isotope fractionation values (Raven et al 1982a, b). The use of carbon isotope fractionation values as a tool for investigating the distribution and activity of CCMs and bicarbonate use versus diffusive CO_2_ entry in algal photosynthesis was to be a persistent theme in John’s later work (see historical sections).

One of the many exciting things about working in John’s lab was the huge range of topics in which he was interested. How could one person know so much about so many things? Over a pint or two of beer in the local pub we would discuss all manner of subjects, and, for instance, I well remember ideas about the factors determining the lower limits for photosynthetic growth being jotted down on beermats, then transcribed by John into two papers on the role of H^+^ permeability of membranes and the consequences of ‘slippage’ reactions (Raven and Beardall 1981, 1982). I started a second postdoc with John at the beginning of 1982 to follow up these ideas, with Kath Richardson joining the lab to work on the same project. However, I moved to La Trobe University in Australia to take up a lecturership in June that year, though we eventually published experimental data on ‘slippage’ (Quigg et al 2006). Before I left John’s lab, Kath, John and I worked on a review of light acclimation strategies in microalgae (Richardson et al 1983), which is well cited to this day.

After I moved to Australia, John and I continued to collaborate, either writing up data we would collect together during his frequent visits ‘down under’. or following up ideas one of us (mostly John it must be said) had for review articles on a range of topics. We continued our collaboration from after I left Dundee in June 1982 until his death in May 2024 and covered topics from the role of primary active transport in algal energetics (Raven and Beardall 2020), via evolution and modulation of CCMs (e.g. Giordano, Raven and Beardall 2005) to the influence of global climate change on plankton (Raven and Beardall 2021). Indeed, it was only a few weeks before his passing that he had finished helping check page proofs of a book edited and contributed to by himself, Mario Giordano, Stephen Maberly and I (Giordano et al. 2024). John was always extraordinarily generous in involving colleagues in collaborative work (the list of his co-authors is enormous!) and his influence on generations of up-and-coming biologists was immense.

Working with John was a great privilege. His lab was a hot-bed of new and exciting ideas (not to mention the eccentricities of a mouldy piece of cheese sitting in a bell jar for years, or the huge pile of reprints on every surface in his office, from which he could pluck any paper needed within a few seconds). His prodigious understanding of a wide range of subjects was amazing but tempered with a sense of humour and a willingness to help colleagues and students. He will be greatly missed.

#### Howard Griffiths

My first memories of John as an undergraduate occurred on a field course at the Dichty Burn, Angus, Scotland (site of later collaborations, e.g. MacFarlane and Raven 1990)), where John, this rather distant, hirsute lecturer, seemed only abstractedly interested in the biota (including us students)… until once we engaged him in conversation, verbal connections, puns and spontaneous jokes ensued. I also remember the excitement of taking notes during his second-year lectures (1973–4) where he explained this “new” chemiosmotic theory, as applied to ATP synthesis. Later, we were much concerned when at the start of the next academic year, John seemed to have wasted away: his doctor, having put him on a diet, had left the practice, and no-one had seen fit to advise John that he was now well beyond an appropriate BMI; such was the single mindedness and determination which characterised his entire academic career!

I was then fortunate to undertake a PhD, supervised by Janet Sprent, with John in an adjoining office, and subsequently a postdoc with John working on oxalate biosynthetic pathways in higher plants, using stable carbon isotope signals (Raven et al 1982a, b). John was a remarkably kind and generous collaborator: he tolerated us folk, who having consumed two or three pints, would head home, whilst he went back to his office to work for another two or three hours. In John’s flexible approach to scince he allowed me to join an expedition to Trinidad for two months in 1981, whose aims were unrelated to my research. At that time Barry Osmond was visiting Dundee (see section by Barry Osmond) and informed us about epiphytic CAM bromeliads. This led in my subsequent career at Newcastle upon Tyne and at the University of Cambridge to collaborations with John, John Beardall and Kath Richardson on algal and isoetid photosynthetic carbon concentrating mechanisms.

In later years John was supportive in providing references and reviews for academic positions and promotions, and we continued to hear of his widespread travels through the postcards and their quirky messages requiring translation from his coded scrawl. Some of us were fortunate to be invited by John to contribute to his retirement symposium in 2008 (Andrews et al 2009), at which many tales were told of the rain-soaked trip to Glen Clova (see images in slideshow pdf, Griffiths and (2024a) Obituary, John Raven, 2024bCE: In addition to seeing him regularly at SEB meetings (he had at one time been President) and the ever-expanding CCM conferences, we kept in touch, and also produced a commentary for Journal of Experimental Botany on carbon partitioning in wheat (Raven and Griffiths 2014), as NOT having been determined from stable isotopes of carbon!

John was gregarious, humorous and loved by those of us who were privileged to have worked with, or be taught by, the great man (Griffiths and (2024a) Obituary, John Raven, , 2024aCE: Please check the year ab automated process, 2024a, b). We had many entertaining evenings at his house in Broughty Ferry (his garden, as described by John Beardall, was an early demonstration of rewilding!). We were all relieved when finally Linda, his wife, took charge, and helped bring some order to the garden and long term support for his continuing academic endeavours. A mark of respect was the response we received upon news of his death. Although he had stipulated there to be no formal funeral, Steve Hubbard and I took it upon ourselves to organise a “Gathering of the Raven Clan”, which drew over 80 folk to Dundee Botanic Garden in July 2024, as well as a raft of tributes and photographs from many unable to attend (Griffiths and (2024a) Obituary, John Raven, 2024aCE: Please check the year ab automated process, 2024b). Amongst the tributes was a telling comment from Alison Roberts (as a former undergraduate and PhD student supervised by John and now a researcher at James Hutton Institute, Dundee): ‘*Whether in a lecture theatre, at home or walking through city centre he was comfortable in his own mind and his own attire. To undergraduates in particular that was a powerful message; young people trying to figure themselves out had a brilliant role model in John and the city largely took him to its heart*’.

To conclude, Raven’s breadth of knowledge was unrivalled across plant and marine sciences, including biophysics and geochemistry. He was an inspirational teacher, and generously supported a legion of postgraduate students and post docs, his ideas providing a platform for their future careers. John was sociable, with a keen sense of humour, which could skewer lofty pretensions, but with the patience to encourage and inform us lesser mortals. Many stories have been told about the seemingly inevitable avalanche of paper across every surface in his office, from which strata a vital document could instantly be recovered; his recall of a specific paper drawn at random from many thousands of diminutive file cards; his pioneering sartorial dress sense. Ultimately, John’s prodigious scientific output represents the mark of a truly original scholar, and his memory will be carried forward by so many of us who were fortunate to have known him.

#### Andrew and Sally Smith

After I finished my Cambridge PhD supervised by Enid MacRobbie in 1965, I had a couple of years in her lab. group as a postdoc and tackled photosynthesis into charophyte cells, using ^14^CO_2_. At the same time, John Raven measured ^14^C uptake in *Hydrodictyon* over a range of solution pH as part of his PhD project with Enid and concluded that at high solution pH there must be HCO_3_^−^ uptake (Raven 1966). I went on to publish two articles showing that there was light-driven HCO_3_^−^ uptake at high pH by four charophytes, *Nitella translucens*, *Chara australis* (aka *C. corallina*), *Nitellopsis obtusa* and *Tolypella intricata*, all of which developed external calcification as a result (Smith 1967, 1968). John published his study in 1968 (Raven 1968). I had got in ahead with my first article because I wanted to tidy up publications before I left Cambridge.

I left for Adelaide in 1967, with my wife Sally, and not long afterwards John left for Dundee. I worked on Cl^−^ transport in *C. corallina* and became convinced that ion transport involves ATP-mediated active H^+^ efflux and passive OH^−^ influx coupled to, and a driver of, Cl^−^ influx (Smith 1970). That paper was published after rejection by the editor of a then high-profile journal on the grounds that he didn’t believe Mitchell’s hypothesis model: a big learning experience on how editors should not behave. I did not favour coupling of Cl^−^ to H^+^, because of the mistaken thought that uptake of ‘HCl’ was dangerous to the plasma membrane – I’ve always regretted that early dismissal, but it was soon corrected as the concept of H^+^-linked ion transport became accepted. It was probably this article that prompted our collaboration, though this may be wrong: he was an avid reader of articles in his many library visits.

I am fairly sure that we next had personal contact when Sally and I had a sabbatical visit to Oxford in 1971. No early written correspondence (if there was any) has survived until March 1972, when John sent the first of the blue aerograms that I have kept (over 20), some of which mention his travels, especially to conferences and planned articles, both joint ones and otherwise. By then he had drafted a joint paper about pH regulation to be presented in the International Workshop on Plant Membrane Transport in Liverpool in 1972, published as Raven and Smith (1973) and he deserves the credit for establishing this main aspect of our collaboration.

John and I were strong believers that intracellular (biochemical) processes alone cannot regulate cytoplasmic pH in plants, whether photosynthetic or otherwise, in the absence of plasma membrane H^+−^ transport: hence our notion of a combined biochemical-biophysical pH–stat (Raven and Smith 1973). One publication acknowledges the contribution of George and J.G. Smith to our “spirited discussions”; they were well-known distillers of whisky that we drank in a long evening session in which John and I drafted the paper during a visit to Dundee.

In 1975 Sally and I spent a sabbatical of some months in Dundee, in which I worked with John on *Hydrodictyon* and Sally (then a young mother) was taken under the wing of Mevin Daft, who introduced her to arbuscular mycorrhizas, later studies of which made her famous. This visit resulted in a seminal paper on chemiosmotic coupling (Raven and Smith 1976a, b) and other publications in 1976.

The list does not reflect all our interests in photosynthesis that led to separate publications. There are two articles with Alan Walker as co-author (Smith et. al. 1977, Raven et al., 1986). By then Alan had become another of my major peer collaborators, with many publications. The other one was Sally, who had encouraged my interests in physiological aspects of mycorrhizal symbiosis, resulting in many joint publications. The list includes John and both Smiths: he already had strong interests in plant evolution.

By the early 1980’s our joint interests in pH regulation had apparently become largely exhausted (note, however, Raven and Smith 1981 & 1982, reflecting John’s ongoing interests in evolution and photosynthesis). Other research and professional activities took over. A later joint article was written in 1990 after a claim that we were wrong in asserting that organic acids (hence H^+^), especially during fermentation, were pH-lowering because of ATP synthesis from ADP, a pH-raising process. Unfortunately, the paper was rejected and the work was never published. Happily, after a long gestation, a substantial review with Sally as co-author was published in 2018 in *New Phytologist* (Raven et al. 2018), a journal in which we had published many of our earlier articles. (Sadly, Sally died in September 2019).

#### Patricia Sanchez-Baracaldo

It is with the deepest respect and admiration that we pay tribute to the remarkable life and work of Professor John Raven. I feel extremely lucky to have known such a brilliant mind, kind soul, and dearest friend.

Professor John Raven was widely regarded as a leading scientist in many research areas, including botany, ecophysiology, and the biochemistry of primary producers spanning plants, algae, and photosynthetic bacteria. I met John at the Royal Society Discussion meeting on “The Global Nitrogen Cycle in the Twenty-First Century” in 2013; John was one of the organisers. During the meeting, we met during breakfast, and we instantly “hit it off,” starting a non-stop conversation about photosynthesis and cyanobacteria. I was at the start of my five-year Royal Society Dorothy Hodgkin Fellowship and felt incredibly lucky to have met a towering figure in the fields of marine biology, plant physiology, and environmental science. We worked together until the week of his passing. John was pleased to hear that I had just submitted the revised version of our manuscript entitled ‘Photosynthetic Primary Production in the Mesoproterozoic’ to *New Phytologist*; I wish I could tell him it has been accepted! John would be delighted to hear that one of the reviewers described it as a ‘monumental review’.

John made seminal contributions to our understanding of algal life forms and their critical role in sustaining marine ecosystems. His investigations into how carbon dioxide, light, and trace minerals interact to limit primary productivity in algae contributed transformative insights, forming the foundation for our knowledge of the complex biogeochemical cycles that underpin ocean health. John was a visionary; he had the ability to go far beyond organism-level processes, extending to wider-scale biogeochemistry, marine and terrestrial ecosystems, paleoecology, and even astrobiology. This breadth of expertise and intellectual curiosity is reflected in his prolific publication record.

John’s legacy extends far beyond his own groundbreaking work. He cared deeply about students, early career scientists, and colleagues, often writing letters of support. I feel incredibly fortunate because he was the best mentor I could have ever wished for. John was so generous with his vast scientific knowledge, as well as his unwavering commitment to rigorous, ethical inquiry. I could ask John anything, and he would get back to me with a detailed and insightful answer on the same day as late as 10 PM. I have archived many of his messages with his responses and attached documents. No artificial intelligence engine could match John’s insights. After my travels to conferences in places he had previously visited, he would remark on plants he had seen with full scientific names. He would also report on plants or flowers he would encounter during his long walks in Dundee—I will really miss this. His generosity of spirit and genuine care for the next generation of scientists have left an indelible mark on the field.

John was involved in leading the pivotal Royal Society review on the state and implications of ongoing ocean acidification (Raven et al 2005a, b, c). This prescient work highlighted the urgent need to address the human-induced environmental changes threatening the delicate balance of marine ecosystems — a call to action that continues to resonate today. John was truly dedicated to safeguarding the natural world for future generations. We should all feel immense gratitude for the indelible mark he has left on science and policy, as well as the many generations of scientists he taught and helped.

John Raven's legacy will forever uphold the highest standards of scientific excellence, collaborative spirit, and unwavering commitment to understanding and preserving the natural systems upon which we all depend. Rest in peace, my dearest friend.

#### Ray Ritchie

I first met John Albert Raven in about 1978. I was already familiar with his work on membrane transport in *Hydrodictyon*. I used his comprehensive cover of what needed to be done in a serious study of membrane transport in an aquatic alga as a guide for my PhD work. Later he was one of my PhD examiners: I passed.

He was true encyclopaedic polymath with a mindboggling photographic memory and was always helpful to colleagues and in particular junior researchers. He was always helpful and constructive rather than discouraging to students. He could and would tell you though if your bright idea was not likely to work but nearly always suggested a better approach.

John Raven was probably one of the best thinkers in plant science in the 2nd half of the twentieth century. He made major advances in experimental plant science and was a major contributor to theoretical biology and the philosophy of science. Much of his later contributions were to insights on photosynthesis and primary production in many different types of habitat, particularly Rubisco, CO_2_ fixation, CO_2_ concentrating mechanisms in algae and terrestrial plants and bicarbonate uptake by aquatic plants. His contributions ranged from the molecular to global scales. He also contributed to nitrogen metabolism in plants.

#### Peter Ralph

When John visited the University of Technology Sydney on many occasions, his presence was a source of inspiration for students, both undergraduate and postgraduate. Before his annual trips down under during the 2010's, I would prepare the new students for his arrival by sharing my signed copy of "Aquatic Photosynthesis." They were meeting one of the co-authors of this seminal book. The postgraduate students who had met him before would come equipped with a list of thought-provoking questions, ready to engage with his profound and encyclopaedic knowledge. Many newcomers were initially surprised by his dry presentation style and attire, but those who took the time to understand him soon learned that the key was to pose a question that steered him into uncharted territory beyond his lecture plan. In those moments, the room would light up as students eagerly attempted to follow his fluid logic. The intellectual reward was always worth it for the postgraduates who prepared thoughtful questions. I fondly remember the experience of co-authoring a particular paper with John on biofuels. Witnessing his mental framework of how a cell's energetic stoichiometry operated was truly fascinating: concepts like the number of protons per mole of ATP relative to the number of moles of NADPH eventually became clear under his guidance. The process involved numerous revisions of the mathematics supporting the stoichiometry to ensure everything aligned perfectly with our narrative. John was a mentor, a deep thinker, and a source of inspiration. His legacy will continue to influence and guide all he met and those who (and will) read his research.

#### John Runcie

John thought deeply about many things. His understanding of the physiology of plants and algae was wide and comprehensive. Once asked a question, John would look at you, he would appear a little blank and remain silent for a while, then start to form a detailed answer which would end quite often with a series of options, depending on various situations one could imagine. Rarely would one be given a yes or no. And always with humour. There was humour in that fact of there being multiple answers or solutions to a problem, and that the world is fundamentally complex.

John liked tea. When asked would he like some, he once replied (and I often recall this statement) that any time is good for drinking tea, and one cannot have too much. So we did drink tea together.

John’s demonstrated a kindness and humbleness perhaps less often seen today. He would speak with anyone without favour, students and professors alike. And would give his time to people when asked for advice or comment. Many students have grown in confidence and humility having spent time with him.

The kilt, often worn by John, was maybe seen by some as an eccentricity, but for him this was a normal thing. I asked him once about it and he said he liked to wear a kilt as it is very comfortable.

**Fig. 7:**
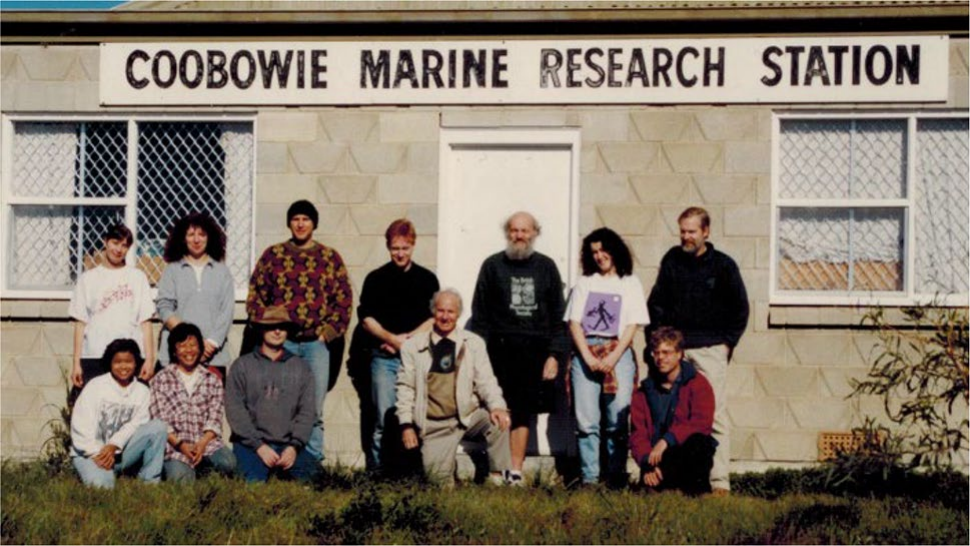
John Raven with colleagues and students at the Coobowie Field Station on the Yorke Peninsula, South Australia, in 2000. John Raven is standing in the middle of the picture in front of the door; on his right, kneeling, is Tony Larkum and on his left is Catriona Hurd and Brian Longstaff, and kneeling, John Runcie. The work was published as Longstaff et al. 2002 (An in situ study of photosynthetic oxygen exchange and electron transport rate in the marine macroalga *Ulva lactuca* (Chlorophyta))

In July 1997, I accompanied Tony Larkum, Anthony Cheshire and his students, John Raven, William Dennison, Catriona Hurd, Todd Kana and Ben Longstaff to the Coobowie Marine Research Station (see Fig. 7), where we deployed Anthony’s submersible respirometer systems and measured oxygen evolution concurrently with electron transport (Longstaff et al. 2002). This was one of the early papers describing a field-based comparison of these two measures. While we beavered away with equipment, John sat in a chair writing a manuscript by hand in a notebook which he later had typed up at the University and published (Raven and Edwards 2004). My recollection is that there was very little crossing out or correcting as John wrote down his thoughts, an example to me of his thorough grasp of the topic at hand, capacity for thought and ability to convey these thoughts in words.

We once caught the bus to St Andrews to collect macroalgae for experiments. John didn't seem interested in driving a car. Sitting in a bus gave us time to talk and think, which was a good thing.

I recall John’s thoughtfulness when I visited him and Linda at their place near Dundee. On more than one occasion I would find a paper placed where I would sit for breakfast, relevant to the previous day’s discussions. John provided me some bench space during that visit in 2003, where I conducted fluorescence experiments on *Plocamium cartilagineum*, *Ulva* sp. and *Fucus vesiculosus* examining the kinetics of recovery of these species from high light exposure. During those early days in my career, I appreciated very much John’s perspective and insights. Most likely many of his comments and suggestions went right over the top of my head. However, some years later conducting deepwater photosynthesis research I looked up John’s papers and realised the depth of knowledge he had for algal physiology. I put this to good use and realised it is not just the data one acquires that is of value but the interpretation of that data in the light of other knowledge. John Raven was a master in this regard.

I will miss John’s humour and good nature, his thoughts, perceptive comments, insights into all things related to algal physiology, comments on other topics. And I will remember that there is always time for tea.

#### Mark Westoby, FAA

I got to know John Raven via the Australia-New Zealand Network for Vegetation Function. This ran from 2006 to 2010, coordinated by myself together with Ian Wright and later Adrienne Nicotra. It held working groups at Macquarie University in Sydney to synthesize data or to move concepts forward. There were 69 working groups in all. Mostly they met for two or three sessions of several days each. John was a participant in two of them. It was in 2006 that I first met him.

WG33 was about allometry and composition. It gave rise to two papers focused on phytoplankton cell size and element stoichiometry (Beardall et al. 2009; Finkel et al. 2009). I was not involved in those projects except for making encouraging noises at the beginning of a meeting and during meal breaks.

In John's other working group (WG3 Nitrogen-Phosphorus) I was a participant myself. At the time (2005–2006) leaf economics was built around carbon. Investment was in units of dry matter, returns on investment were in units of photosynthate. Obviously nitrogen and phosphorus are also meaningful investments into leaves, so the initial idea was to try to advance how we think about nutrient economy in relation to carbon economy.

It went along in a way that was fairly typical of working groups. On the first day everyone gave a brief overview of what they were most interested in. There were many really interesting bits, along with evident disagreements here and there. At the end of the afternoon a provisional list of possible projects was built. A meal out for the group that evening re-ran some of the arguments in a cheerful setting. The next morning it began to emerge where a cluster of people had a definite idea and wanted to push it forward together. Others were just going to comment from the margins.

In this setting, John Raven was a remarkable contributor. His grasp of physiological detail was astonishing; he had recall of literature across a startlingly wide range, plus apparently encyclopaedic knowledge of biochemical pathways and associated ATP costs.

One product that emerged was an overview in Trends in Ecology and Evolution (Lambers et al. 2008) about how nutrient uptake strategies shifted through the course of soil ageing, for example in chronosequences on dunes. The working group had successfully brought together Hans Lambers's enthusiasm for cluster roots, Sally Smith's knowledge of mycorrhizas, Gus Shaver's ecosystem science and John Raven's physiology. Another product was a Tansley Review of the costs of phosphorus uptake by different mechanisms and from different soil pools, and the consequences of those costs for coexistence of different uptake strategies (Raven et al. 2018). This paper evolved over twelve years. It became known among the authors as the "hairy mammoth" manuscript, being large as well as rife with minds failing to meet between the different authors. Through much of its evolution it covered nitrogen as well as phosphorus uptake costs, but the nitrogen was eventually dropped, partly because of unresolved questions but mainly in order to shrink it to something within the normal range of paper sizes. Through all of this John Raven long-sufferingly took on the lead author role. He navigated patiently among co-authors with divergent priorities, and later on through review and editorial comments.

Aside from much emailing to and fro over these 12 years, I saw John for supper from time to time when he visited Sydney. What was he like as a person? – he was just a really nice man! He always seemed to listen to the concerns of others more so than urging forward an agenda of his own. Friendly, mild-mannered, constructive. It was helpful to be aware of his giant stature as a researcher, because if you were trying out a wild idea on him, you actually had to push quite hard before he would start to tell you what was wrong with it. I'm really sorry that he’s lost to us.

#### Tony Larkum

“Into the Voids”. I always thought that was a provocative title for one of John’s papers on vacuoles! Or maybe you prefer another poignant, Shakespearean title: “Put out the light and then put out the light” (Raven et al. 2000). Sadly, John has gone into the void and I miss him very much!

I first met Albie, as we called John Albert, in the lab of Enid MacRobbie in 1964, when I was testing out for a UK Agricultural Research Council Research Fellowship in the Botany School, Cambridge. What an extraordinary character he was! Always cooking up a new experiments on *Hydrodictyon,* while writing out innumerable card references for journals that I had never heard of!

I came out to Australia in 1969 and John went to Dundee. But we kept up a constant conversation and John came out to Australia many times, where he worked with Andrew Smith and Alan Walker in Sydney, and Graham Farquhar in Canberra, and in his later days with Hans Lambers in Perth. Ground-breaking papers emerged from all these collaborations (Smith and Raven 1979; Raven and Farquhar 1990; Raven et al 2018).

I well remember the troubles caused by the success of the Internet in the late 80 s. By this time John had accumulated an office-full of references on cards. It was with great difficulty that he was persuaded to employ a secretary to transfer these onto a computer! And I am not sure that it ever fully had his confidence!

Later he began a series of visits to Australia, becoming a Visiting Fellow at Macquarie University with Mark Westoby in the late 90 s. This was when John and I began to publish together (Raven et al 2013; Larkum et al 2017, 2018)—papers well down the citation index of his publications! But what we all remember is his transition to kilts and then to skirts. Sometime around 2000, I remember walking down Rundle Mall in Adelaide; John was wearing a provocative skirt and a flousy shirt, and I was quite worried that we would be accosted by the lads there, who were well-known for beefing-up what are today known as the LBGBTIQ community. Later John came as a visiting fellow to the University Technology Sydney, often wearing similar dress. But we never did get to the bottom of it!

In April, 2024, I was writing up a paper with John, Duncan Fitzgerald and Maria Ermakova on organisms with only Photosystem I: I sent John the latest draft—and wondered why I did not receive a reply the next day! RIP! Dear Friend.

## Data Availability

No datasets were generated or analysed during the current study.
